# Deep learning-based elaiosome detection in milk thistle seed for efficient high-throughput phenotyping

**DOI:** 10.3389/fpls.2024.1395558

**Published:** 2024-07-26

**Authors:** Younguk Kim, Alebel Mekuriaw Abebe, Jaeyoung Kim, Suyoung Hong, Kwanghoon An, Jeehyoung Shim, Jeongho Baek

**Affiliations:** ^1^ Gene Engineering Division, National Institute of Agricultural Sciences, Rural Development Administration, Jeonju, Republic of Korea; ^2^ Genomics Division, National Institute of Agricultural Sciences, Rural Development Administration, Jeonju, Republic of Korea; ^3^ EL&I Co. Ltd., Hwaseong, Republic of Korea

**Keywords:** milk thistle, elaiosome, deep learning, object detection, Detectron2, phenotyping

## Abstract

Milk thistle, *Silybum marianum* (L.), is a well-known medicinal plant used for the treatment of liver diseases due to its high content of silymarin. The seeds contain elaiosome, a fleshy structure attached to the seeds, which is believed to be a rich source of many metabolites including silymarin. Segmentation of elaiosomes using only image analysis is difficult, and this makes it impossible to quantify the elaiosome phenotypes. This study proposes a new approach for semi-automated detection and segmentation of elaiosomes in milk thistle seed using the Detectron2 deep learning algorithm. One hundred manually labeled images were used to train the initial elaiosome detection model. This model was used to predict elaiosome from new datasets, and the precise predictions were manually selected and used as new labeled images for retraining the model. Such semi-automatic image labeling, i.e., using the prediction results of the previous stage for retraining the model, allowed the production of sufficient labeled data for retraining. Finally, a total of 6,000 labeled images were used to train Detectron2 for elaiosome detection and attained a promising result. The results demonstrate the effectiveness of Detectron2 in detecting milk thistle seed elaiosomes with an accuracy of 99.9%. The proposed method automatically detects and segments elaiosome from the milk thistle seed. The predicted mask images of elaiosome were used to analyze its area as one of the seed phenotypic traits along with other seed morphological traits by image-based high-throughput phenotyping in ImageJ. Enabling high-throughput phenotyping of elaiosome and other seed morphological traits will be useful for breeding milk thistle cultivars with desirable traits.

## 1 Introduction

Milk thistle [*Silybum marianum* (L.) Gaertn.] is a biennial or annual plant belonging to the Asteraceae family. It is one of the widely known medicinal plants used as a supportive treatment for liver diseases (Abenavoli and Milic, 2017; Gillessen and Schmidt, 2020; Marceddu et al., 2022). Milk thistle is currently a reliable source of silymarin, a flavonoid complex that is linked with the therapeutic effects of milk thistle. Although silymarin is present in all parts of the plant, the seeds contain the highest amount (Lv et al., 2017). Most of the previous studies focused on the chemistry, genetics, and bioactivity of this important plant, while phenotyping of morphological traits received less attention (Bijak, 2017).

Seed morphological traits provide information for biodiversity analysis of germplasm collections and genotypic discrimination. Since seeds are relatively easy to handle and store, using seed traits for genetic diversity analysis is advantageous (Dong et al., 2016, 2023). The measurements of various seed morphological traits and correlation analysis can be used in breeding to improve seed yield and quality and to understand the genetic basis of trait variation (Finch-Savage and Bassel, 2016). However, effective and accurate phenotyping of seed morphological traits needs the measurement of a larger number of samples. Manual measurement of seed morphological traits is limited to few parameters and is time-consuming, labor-intensive, and error-prone. The development of digital phenotyping technologies is greatly improving these limitations and accelerating plant phenotyping tasks (Furbank and Tester, 2011; Yang et al., 2020; Song et al., 2021). To enable image-based high-throughput phenotyping of seed morphological traits, different tools have been developed including SmartGrain (Tanabata et al., 2012), GrainScan (Whan et al., 2014), SeedExtractor (Zhu et al., 2021), and AIseed (Tu et al., 2023), which greatly improved the challenges of seed high-throughput phenotyping. Image analysis has been increasingly used to extract features of seed images in different crops (Baek et al., 2020; Ropelewska and Rutkowski, 2021; Dong et al., 2023).

Deep learning algorithms are powerful for analyzing complex datasets and give fast and robust results. Although deep learning algorithms can work with any type of data, some algorithms can be best suited to perform specific tasks, and the selection of a suitable model that better works for your specific task is essential (Sarker, 2021). Some of the commonly used deep learning algorithms include Convolutional Neural Network (CNN), Recurrent Neural Network (RNN), Long Short-Term Memory Network (LSTM), Radial Basis Function Network (RBFN), Multilayer Perception (MLP), Deep Belief Network (DBN), and Auto-encoders. In the past few years, there has been advancement in deep learning algorithms, leading to its widespread application in agriculture for crop monitoring, yield prediction, weed and pest detection, disease detection, nutrient deficiency detection, and crop identification by digital image processing (Meshram et al., 2021; Farjon et al., 2023; Liu and Zhang, 2023).

Moreover, deep learning algorithms can be used for fruit detection and counting, which is crucial for yield estimation (Afonso et al., 2020; Liu et al., 2020). Similarly, machine learning algorithms have been recently applied in seed analysis for seed counting, phenotyping, quality assessment, defect detection, and germination detection (Genze et al., 2020; Farjon et al., 2023). A convolutional neural network and transfer learning-based high-throughput soybean seed phenotyping method was proposed by Yang et al. (2021), which automatically generates synthetic labeled images to reduce the cost of image labeling. Semi-automatic image labeling was also implemented in RustNet (a neural network-based image classifier) for high-throughput detection and classification of wheat stripe rust disease in the field. Predictions of the previously trained model with manual correction were used for retraining the model, thereby increasing the labeling efficiency and gradually improving the accuracy of the model (Tang et al., 2023).

Milk thistle contains a fleshy structure attached to the seeds known as elaiosome, which is rich in lipids and proteins. Elaiosomes are found in many plant species and have many colors, shapes, and sizes (Sasidharan and Venkatesan, 2019). Phenotyping of elaiosome will enable us to better understand and dissect the metabolome profile of milk thistle seed. Moreover, the phenotypic information can be used to study the genetic basis of elaiosome variation in different plant species.

Despite the availability of various image-based methods for high-throughput phenotyping of different seed traits, elaiosome phenotyping cannot be performed using only existing image analysis tools. This is because the white stripe on the seed coat of milk thistle resembles the elaiosome color, which makes it difficult for segmentation based on color threshold. Such limitations need the application of deep learning algorithms, which can effectively segment the area of interest by learning the deep features of images (Minaee et al., 2022; Li et al., 2023). In this study, a deep learning model, Detectron2, was trained for elaiosome detection and segmentation in milk thistle seed to enable its high-throughput phenotyping.

## 2 Materials and methods

### 2.1 Plant materials and image acquisition

A collection of 397 milk thistle germplasms received from EL&I Co., Ltd. (https://www.elniseed.com) was used in this study. Seed images of the germplasm were acquired using a digital camera (Sony α6000, 6000 × 4000 resolution; Sony, Tokyo, Japan) and saved in the JPG (Joint Photographic Experts Group) format. Images were captured by spreading approximately 100 seeds of each line on a blue background without contact between seeds. Then, the individual seeds were segmented and saved as a single image file, making a total of ~39,700 individual seed images. Images were captured at Phenome studio, Plant Phenome Research Center, National Institute of Agricultural Sciences, Rural Development Administration, Jeonju, Republic of Korea.

### 2.2 Experimental operation environment

The processing unit consists of Intel^®^ Core™ i7–8700 CPU and NVIDIA GeForce RTX3090 GPU with 62 GB of memory. The environment for deep learning-related procedures includes Python v3.7 and PyTorch v1.8, which were operated in Ubuntu 20.04 operating system.

### 2.3 Image preprocessing

**Figure 1:**
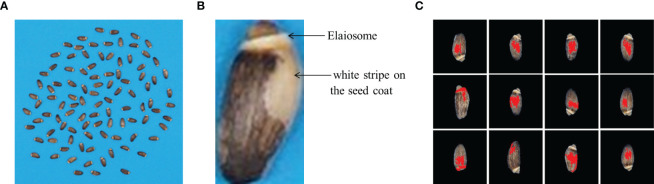
Using only image processing for elaiosome segmentation. **(A)** Raw images of milk thistle seeds; 100 seeds of each line were captured together. **(B)** Elaiosome region and the seed coat white pattern with similar colors, making it difficult to segment elaiosome region using image processing. **(C)** Results of image processing for elaiosome segmentation. These sample images indicate only using image processing based on color thresholding to segment elaiosome region in milk thistle seed.

During image acquisition, 100 seeds of each line were captured at once. The acquired original image was too large to recognize elaiosome using the Detectron2 model. Therefore, individual seeds were segmented and saved as a single image file, making a total of ~39,700 seed images. From the original image, the blue background and the seed portion were processed and separated based on YUV color using ImageJ (Schneider et al., 2012; Baek et al., 2020). Seed images were resized into sizes of 170 × 170 pixels in order to adjust to the requirements of the deep learning model. Background removal and foreground extraction were performed to reduce the overall noise in the image. For model training, images were prepared with three backgrounds (black, blue, and white) in order to enhance the robustness of the model to detect in varied conditions. Segmentation of elaiosome only using image processing based on color thresholding was not successful due to the interference of the white stripe on the seed coat, which resembles the color of the elaiosome (**Figure 1**).

### 2.4 Image augmentation

**Figure 2:**
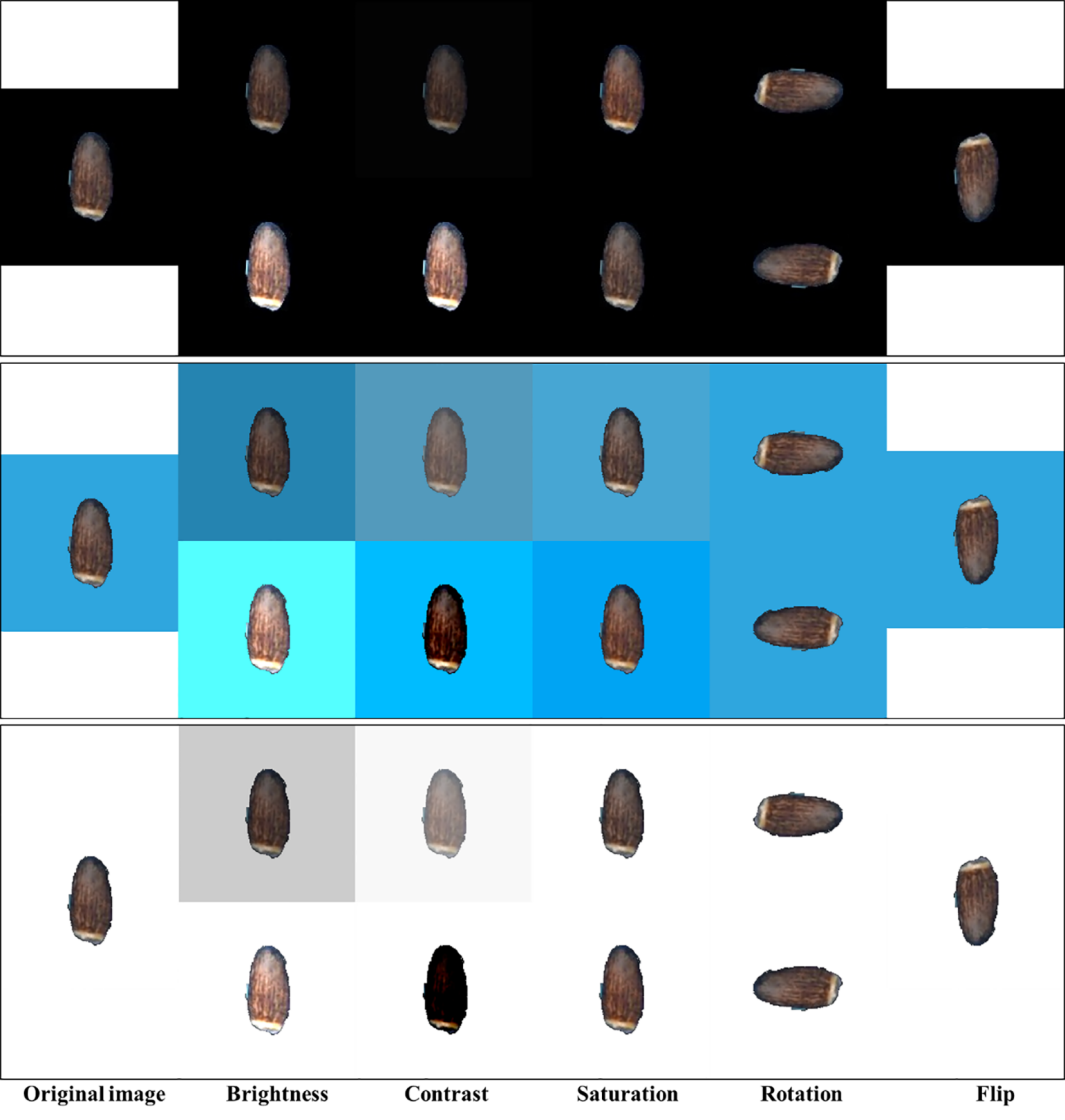
Sample images of different image augmentation techniques. Only minimum (top) and maximum (bottom) value augmentations are shown for each black, blue, and white background.

Using an adequate number of sample size is vital to obtain robust results from the deep learning model. The model trained with a large sample size has stronger generalization ability. Data augmentation is a successful method used to deal with a limited number of data for deep learning. It includes generating new training images from the original dataset by applying visual and spatial transformations before being used in training (Shorten and Khoshgoftaar, 2019; Mumuni and Mumuni, 2022). The Detectron2 model provides various types of augmentation techniques. We applied default augmentation methods provided by Detectron2 such as random augmentation of brightness, contrast, saturation, rotation, and flipping to our datasets to increase the diversity of the input images (**Figure 2**). Brightness or the amount of hue was adjusted with a random value within the range of 0.8 to 1.8, which means that the augmented image can have a brightness of anywhere between 0.8 (darker) and 1.8 (brighter) times compared to the original image. Contrast is the amount of luminance and was adjusted with a random value within the range of 0.6 to 1.8. Saturation indicates the purity of the color and was adjusted with a random value within the range of 0.8 to 1.4. Such color space augmentations will help the model learn different lighting and color conditions to improve the robustness and generalization of the model. Geometric transformations, rotation and flip, were performed by rotating the image by a random angle within the range of −90° to 90° and vertical flip with a 40% probability, respectively. These augmentations can help the model to learn to handle different orientations and positions of elaiosome in the image.

### 2.5 Detectron2 model architecture

**Figure 3:**
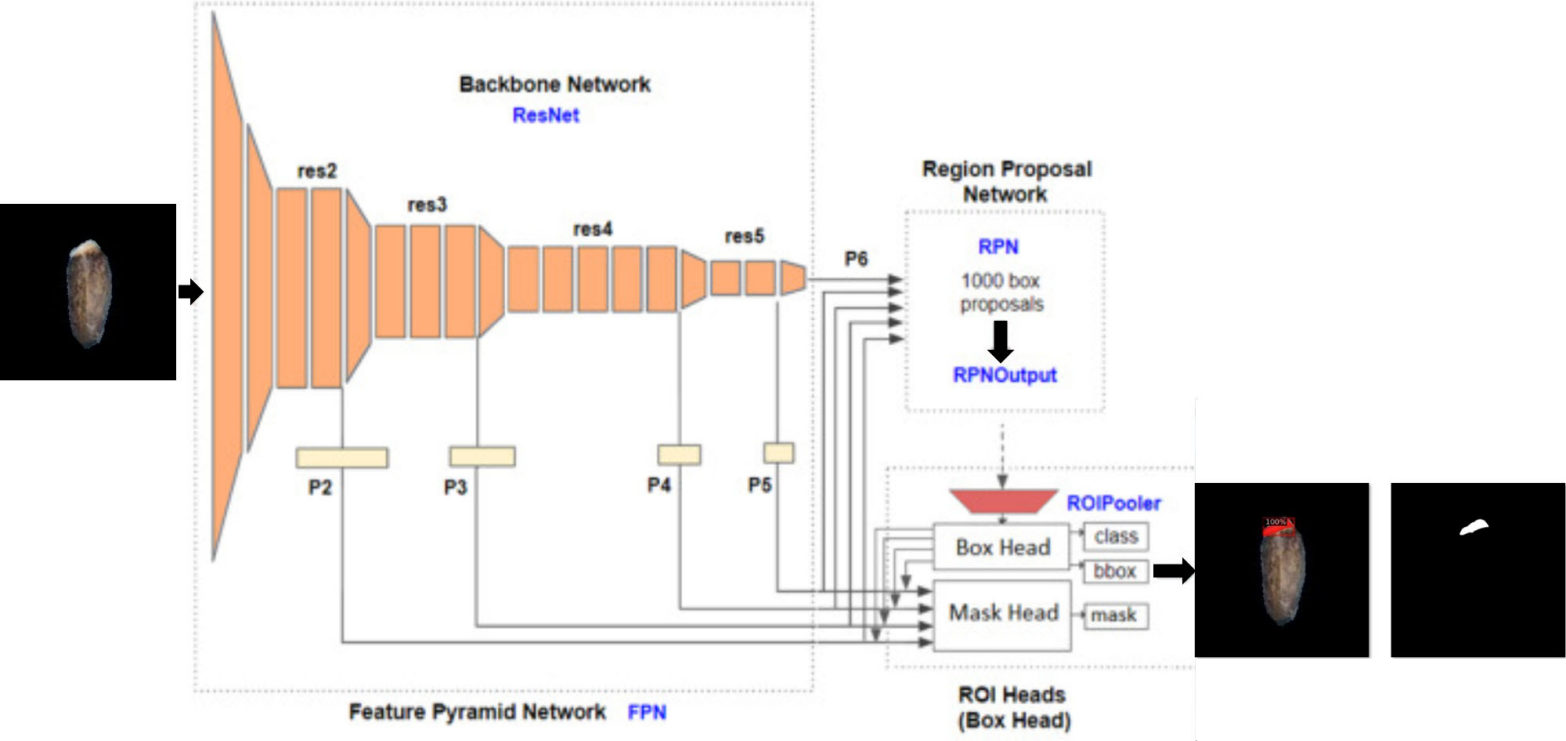
Detectron2 model architecture.

Detectron2 is an object detection model released by Facebook’s AI research team and was developed on top of the PyTorch deep learning framework. It is the second version of the Detectron framework and provides object detection and segmentation algorithms. Although it was initially trained using images from daily life, Detectron2 allows transfer learning (enabling the rapid retraining of the model using custom datasets from a different domain) (Wu et al., 2019). It combines different deep learning models for object detection such as Faster R-CNN, Mask R-CNN, RetinaNet, and DensePose. This model consists mainly of three blocks: Feature Pyramid Network (FPN), Region Proposal Network (RPN), and Region of Interest (ROI) heads. FPN as a backbone network is responsible for extracting features from input images during the learning process. Features extracted at each res stage (convolution blocks of “ResNet”, the backbone network) are used as input for RPN and ROI heads. The RPN is used to specify the location of the candidate bounding box with a confidence score in the input feature. ROI head block consists of a box head and a mask head. The final outputs of the ROI block are predictions of the class (object level classification), bounding box (localization), and segmented mask of objects (pixel level classification) from the characteristics received from FPN and RPN (**Figure 3**) (Wu et al., 2019; Ackermann et al., 2022).

### 2.6 Image labeling and model training

Detectron2 offers more accuracy and flexibility and supports various backbone architectures, making it a choice for accurate and fast object detection. It was written in the PyTorch framework, which is easy to customize and extend. Here, we used Detectron2 for accurate detection and segmentation of elaiosome in milk thistle seeds. In deep learning, the image labeling step is a time-consuming and labor-intensive task because the model training requires large amounts of labeled data. To reduce the time and labor power required for image labeling, we employed image processing and deep learning to enable semi-automatic image labeling of elaiosome in milk thistle seeds. Few seeds that were precisely detected as elaiosomes using image processing were selected and labeled using ImageJ in the form of segmentation. These images were used to train the first elaiosome detection Detectron2 model. Then, this model was used to predict elaiosome in the entire dataset, and the correct prediction segmentation masks were manually selected and used as labeled images for retraining the model.

**Figure 4:**
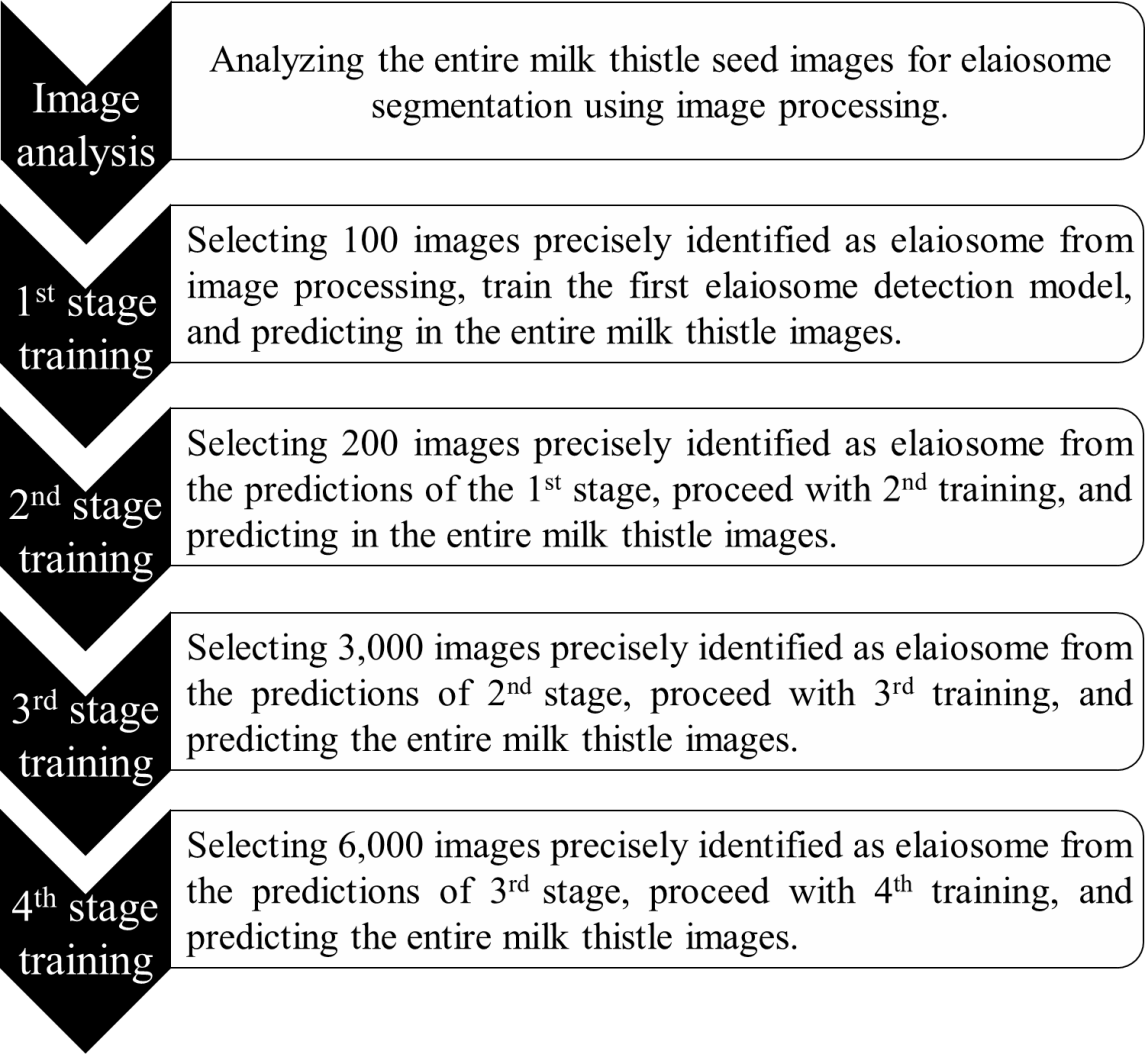
Overview of model development for elaiosome detection. After each training stage, the model was used to predict elaiosome in the entire milk thistle images (~39,700 images). The training set for the next stages was manually selected from the prediction results of the previous stage and used for retraining the model.

The dataset was divided into a ratio of 8:1:1 for training, validation, and testing. Before feeding to the model, images were prepared in COCO (Common Objects in Context) format (JSON file, containing all the characteristics of the images including size, bounding box coordinates, and labels of the box). To improve the prediction accuracy of the model, the training was repeated using the correctly predicted images with manual checking from the previous training stages. The second training was conducted using the predicted images (n = 200) from the results of the first trained model. Similarly, the third and fourth training were repeated using the correctly predicted images in stage 2 (n = 3,000) and stage 3 (n = 6,000) (**Figure 4**). Semi-automatic labeling, i.e., selecting and using the prediction results of the previous stage as input for retraining the model, helps to obtain an adequate number of labeled data easily and saves much time and labor because it takes significantly less time to verify if an automatically suggested label is correct than to manually label from scratch. During the training process, a learning rate of 0.000005 and a batch size of 40 were used.

### 2.7 Evaluation of the model performance

Model evaluation is an essential aspect of deep learning. The prediction accuracy of the elaiosome detection model was evaluated using various evaluation metrics including precision, recall, F1-score, specificity, and accuracy. The results of the model prediction are classified into true positive, false positive, true negative, and false negative. The ground truth data of the test set were used to derive positive and negative counts and were calculated using the following formula (Tang et al., 2023).


$$\text{- Accuracy}=\frac{\text{True Positve}+\text{True Negative}}{\text{True Positive}+\text{True Negative}+\text{False Positive}+\text{False Negative}}$$

$$\text{- Recall}=\frac{\text{True Positive}}{\text{True Positive}+\text{False Negative}}$$

$$\text{- Specificity}=\frac{\text{True Negative}}{\text{True Negative}+\text{False Positive }}\;$$

$$\text{- Precision}=\frac{\text{ True Positive}}{\text{True positive}+\text{False positive}}$$

$$\text{- F}1\text{ score}=\frac{\;2*\text{Precision}*\text{Recall}}{\text{Precision}+\text{Recall}}$$

$$\text{- Average Precision}\left(\text{AP}\right)=\sum_{n=0}\left(Recall_{n+1}-Recall_{n}\right)\rho interp\left(Recall_{n+1}\right)$$

∘ printerp(Recalln+1)=maxr¯:r¯≥rn+1ρ(r˜)

∘ where $\rho \left(\tilde{r}\right)$ is measured precision at recall $\tilde{r}$


$$\text{- Mean Average Precision }\left(\text{mAP}\right)\;=\sum_{n=1}^{cn}AP_{n}$$

∘ cn is number of class

$\mathrm{\circ A}P_{n}$ is measured AP about class n.

Intersection over Union (IoU) is a basic evaluation metric that measures the similarity and diversity of two sets. IoU is a good measure of the amount of overlap between two bounding boxes or segmentation masks. The ratio of the area of overlap to the area of union was calculated between the ground truth (gt) and the prediction results for each stage of the training. A higher IoU indicates a more accurate detection of the model. To assess the prediction results of the models at different stages (first, second, third, and fourth stages), we compared the predicted images between these stages using the Jaccard similarity coefficient. To calculate similarity, we used the predicted mask IoU of the fourth stage with the first, second, and third stages.


$$\text{- Boundingbox}\_\text{IoU}=\;\frac{Area\left(Box_{gt\;}\cap \;Box_{predict}\right)}{Area\left(Box_{gt\;}\cup \;Box_{predict}\right)}$$

$$\text{- Mask IoU}=\;\frac{Area\left(Mask_{gt\;}\cap \;Mask_{predict}\right)}{Area\left(Mask_{gt\;}\cup \;Mask_{predict}\right)}$$

$$\text{- Similarity}=\frac{\text{ ycount}}{\text{ycount}+\text{ncount}}$$

$$\text{ ycount}+1\text{ if }\frac{\text{Area}\left(\text{predictMask}4\text{th }\cap \;\text{predictMaskn}\right)}{\text{Area}\left(\text{predictMask}4\text{th }\cup \;\text{predictMaskn}\right)}>0.9,\;\text{n =}3^{rd},\;2^{nd},\;1^{st}\text{stages}$$

$$\text{ ncount}+1\text{ if }\frac{\text{Area}\left(\text{predictMask}4\text{th }\cap \;\text{predictMaskn}\right)}{\text{Area}\left(\text{predictMask}4\text{th }\cup \;\text{predictMaskn}\right)}\;\leq \;0.9$$

### 2.8 Image-based high-throughput phenotyping of milk thistle seed

Seed morphological traits were analyzed for 397 milk thistle germplasm by high-throughput method. Seed images of milk thistle were processed and analyzed following our previously developed pipeline for high-throughput phenotyping of seed morphological traits using ImageJ (Baek et al., 2020). In total, 10 seed traits including area, perimeter, major axis, minor axis, solidity, circularity, roundness, solidity, aspect ratio (AR), elaiosome area, and the ratio of elaiosome area were measured for a total of ~39,700 seeds using image-based high-throughput phenotyping. For the analysis of the elaiosome area, predicted segmentation mask images were used as inputs. One hundred seeds of each line were analyzed by high-throughput method, and an average was calculated.

## 3 Results and discussion

### 3.1 Elaiosome detection using Detectron2

Detection of elaiosome using deep learning will help in high-throughput phenotyping of milk thistle seed. Enabling elaiosome phenotyping will be an important input for better understanding and dissection of the genetics and metabolomics of milk thistle seed. Image processing allowed us to select some images precisely showing segmentation of elaiosome region. Such images were selected and automatically labeled using ImageJ and used for training the first elaiosome detection Detectron2 model. The labeling was performed as a form of segmentation in ImageJ, enabling automatic analysis of labeling processes in the following steps.

Initially, we used only 100 images to train the first elaiosome detection model. We used this model to predict elaiosome in the entire image dataset (~39,700 images). Two hundred images were manually selected from the correct predictions of the previous model and used to retrain the model. The third model was trained using 3,000 manually selected images from the prediction results of the second model. Similarly, the fourth model was trained using 6,000 selected images from the prediction results of the third model, which greatly improved the prediction accuracy of the updated model. The developed method, which uses few manually labeled images for training the first detection model and retraining the model using correctly predicted images of the previous model, will greatly improve the challenges of the image labeling process.

**Figure 5:**
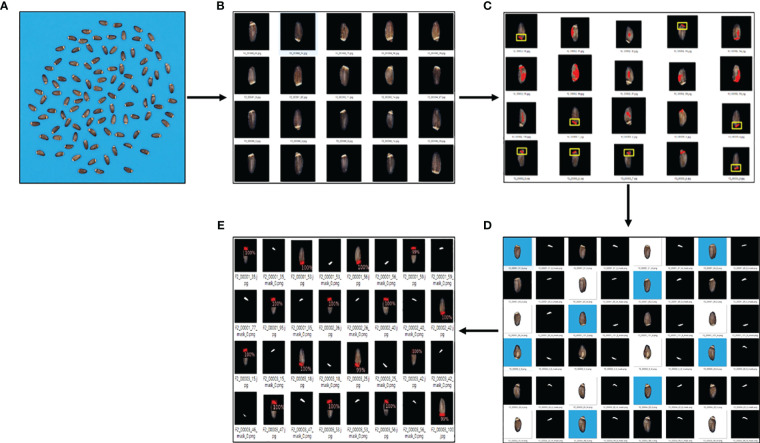
Overview of the elaiosome detection using Detectron2. **(A)** Raw images of milk thistle seeds; 100 seeds of each line were captured together. **(B)** Image preprocessing (individual seed segmentation, background removal, and resizing). **(C)** Results of using only image processing for elaiosome segmentation. Images indicated in yellow box were precisely identified as elaiosome, while the other images show incorrect results. **(D)** Images precisely identified as elaiosomes were selected (n = 100), automatically labeled in the form of segmentation, and used to train the first elaiosome detection model. Images with three backgrounds (black, blue, and white) were used for training to enhance the robustness of the model. **(E)** Prediction results of Detectron2 indicating bounding box and the corresponding segmentation mask images of elaiosome.

Previously, high-throughput soybean seed phenotyping using CNNs and transfer learning was shown to be effective for large-scale accurate quantification of morphological parameters. This method uses synthetic image generation and augmentation to train instance segmentation networks for high-throughput soybean seed segmentation. It significantly decreases the cost of manual annotation and facilitates the preparation of training datasets. The use of transfer learning can reduce computing costs by fine-tuning pre-trained model weights (Yang et al., 2021). Mask R-CNN and YOLO, two popular deep learning models combined with techniques like domain randomization and transfer learning, have also shown promising results for seed phenotyping. Domain randomization and transfer learning approaches were applied to alleviate the need for large amounts of training data, which is often a bottleneck in phenotyping. Domain randomization is a technique used to improve the robustness of deep learning models by training them on a diverse set of synthetic images with randomized backgrounds, lighting conditions, and other variations. This helps the model generalize better to real-world images, which can have significant variations compared to the training data. Transfer learning, in contrast, involves using a pre-trained model (e.g., on a large dataset like ImageNet) as a starting point and fine-tuning it on the specific task and dataset of interest, which can improve performance with limited training data (Margapuri and Neilsen, 2021). Our approach simplified the labeling process to obtain an adequate number of training data using real milk thistle images. The model was able to predict the elaiosome and give an output of class, which is only one in this case, bounding box, and the corresponding mask images of the elaiosome region (**Figure 5**).

**Figure 6:**
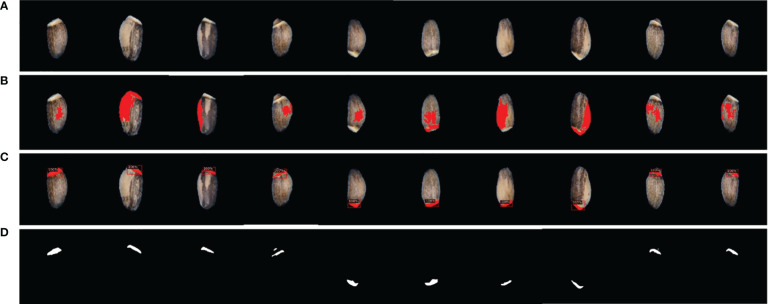
Visualization of elaiosome detection using Detectron2. **(A)** Original seed image. **(B)** Representative images of incorrect elaiosome segmentation by image processing. **(C)** Predicted bounding box of the corresponding images showing the precise detection of elaiosome by Detectron2. **(D)** Predicted segmentation mask images of elaiosome by Detectron2.

Our study developed a deep learning approach based on Detectron2 for the detection and segmentation of elaiosome from milk thistle seed for the first time. Our deep learning approach was able to precisely predict and segment elaiosome in images where only image processing did not succeed (**Figure 6**). The resulting mask images of elaiosome were used as inputs for quantitative analysis of elaiosome phenotypes using ImageJ. This brings a new trait of milk thistle seed under consideration, which was previously difficult to measure.

### 3.2 Evaluation of the model performance

The performance of a deep learning model can be influenced by various factors including the quality and quantity of the training dataset, the model architecture, hardware and software libraries, and hyperparameters (learning rate, batch size, and number of iterations) (Kamilaris and Prenafeta-Boldú, 2018). The performance of Detectron2 in detecting elaiosome in milk thistle seeds was evaluated using average precision (AP), loss, and accuracy curves.

**Figure 7:**
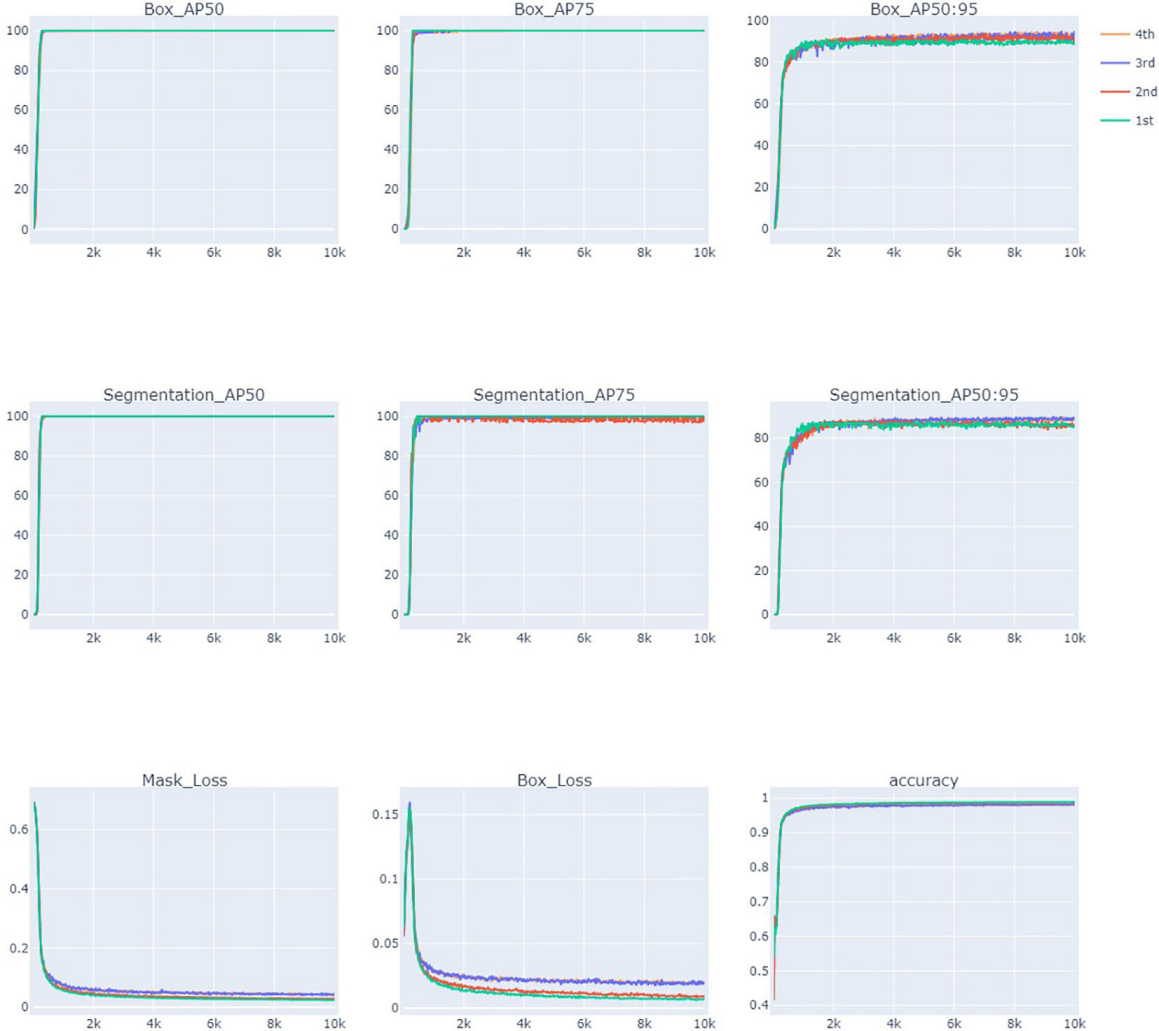
Evaluation of elaiosome detection model Detectron2 using average precision (AP), loss, and accuracy curves during 10,000 iterations.

The results of the AP, loss, and accuracy curves for 10,000 iterations are presented in **Figure 7**. AP and accuracy increased as the number of iterations increased for all training stages. AP measures the quality of a detection algorithm across different confidence levels of recall and is calculated as the area under the precision-recall curve. It is calculated using multiple mask IoU threshold levels from 0.5 to 0.95 with a 0.05 increment. AP_50_ is the AP value when the IoU is 0.5 (at least 50% overlap between the ground truth and model predicted box or mask) and is a relatively low threshold. AP_50_ reached a maximum of 100% for the bounding box and more than 90.15% for the mask. AP_75_ indicates AP when the IoU is 0.75, which is the stricter threshold, as it requires more correct matching. AP_75_ for the bounding box is more than 99%, and for the mask, it is more than 81.1% for all the training stages, indicating the highest accuracy of the model. AP_50:95_ is calculated by averaging all the IoU thresholds from 0.5 to 0.95. The average precision of the model after 10,000 iterations reached 93.9% (box_AP) and 89.694% (Mask_AP). The detection accuracy of the test datasets in all stages showed more than 90% and generally showed stable patterns when the number of iterations patterns was more than 1,000. The loss value decreases as the number of iterations increases and finally approaches the lowest values of 0.018 (Box Loss) and 0.040 (Mask Loss) in the fourth stage (**Table 1**, **Figure 7**). These results indicate that the adopted model learns efficiently and has the potential to produce the desired outcomes.

**Table 1:** Summary of evaluation metrics for the performance of elaiosome detection model.

Training stage	Box_Loss	Mask_Loss	Accuracy	Bounding Box			Mask		
				AP_50_	AP_75_	AP_50:95_	AP_50_	AP_75_	AP_50:95_
1st	0.006	0.102	0.955	100	100	76.585	90.149	81.089	54.4
2nd	0.007	0.112	0.949	100	100	76.053	100	88.22	60.533
3rd	0.022	0.136	0.937	100	99.01	79.137	99.007	94.817	63.964
4th	0.018	0.04	0.983	99.995	99.01	93.907	99.995	99.995	89.694

The prediction performance of the deep learning model was also evaluated based on accuracy and error rates. They indicate the relationship between the actual and predicted values of the proposed model. Accuracy is a measure of how well a model correctly predicts a class label, while the error rate indicates the proportion of incorrect predictions by the model. For a better understanding of the model, performance evaluation metrics such as precision, recall, specificity, and F1-score were also used to evaluate it. The true and false counts were obtained within training, validation, and test datasets using a minimum score threshold greater than 0.5. The results indicate that the elaiosome prediction accuracy of the model is 99.9% for training, validation, and test datasets (**Table 2**). Mask R-CNN was employed for elaiosome detection and segmentation for comparative analysis with our method. The evaluation metrics show almost similar patterns in both models (**Table 2**).

**Table 2:** Performance comparison of Detectron2 and Mask R-CNN for elaiosome detection.

Dataset	Algorithm	Detectron2				Mask R-CNN			
	Training stage	1st	2nd	3rd	4th	1st	2nd	3rd	4th
Training	Precision	0.99519	0.99542	0.99298	0.98874	0.978329	0.974487	0.968505	0.984312
	Recall	0.95089	0.95005	0.91929	0.93423	0.959738	0.961088	0.957945	0.93943
	Specificity	0.99997	0.99997	0.99996	0.99993	0.999843	0.999819	0.999785	0.999896
	F1-score	0.97254	0.97221	0.95472	0.96071	0.968945	0.967741	0.963196	0.961347
	Accuracy	0.99961	0.99961	0.9994	0.99948	0.99955	0.999542	0.999498	0.999481
Validation	Precision	0.97476	0.96633	0.99355	0.98802	0.95097	0.932799	0.95894	0.978402
	Recall	0.93487	0.9523	0.9199	0.93269	0.934974	0.960577	0.947996	0.930996
	Specificity	0.99982	0.99976	0.99996	0.99992	0.999634	0.999509	0.999722	0.999858
	F1-score	0.9544	0.95927	0.95531	0.95956	0.942904	0.946484	0.953437	0.95411
	Accuracy	0.99933	0.99943	0.99941	0.99946	0.999146	0.999234	0.99937	0.999385
Test	Precision	0.95905	0.97012	0.98606	0.98525	0.947285	0.954069	0.954173	0.976927
	Recall	0.926	0.88811	0.91407	0.93174	0.891297	0.889488	0.943334	0.930324
	Specificity	0.99972	0.99981	0.99991	0.9999	0.999645	0.999698	0.999685	0.999847
	F1-score	0.94224	0.9273	0.9487	0.95774	0.918438	0.920648	0.948723	0.953056
	Accuracy	0.99919	0.99903	0.99932	0.99943	0.998875	0.998924	0.999295	0.999368

Recently, the application of Detectron2 for object detection has gained attention due to its speed and accuracy compared to other deep learning algorithms. For instance, Wang et al. (2023) demonstrated that a system built on Detectron2 outperformed YOLO v8, the latest version of the YOLO model in terms of both speed and accuracy for segmentation of pods in Rapeseed. Detectron2 was also found to be more effective for forest fire detection than other detection algorithms, such as Dilated CNN, AlexNet, Faster R-CNN, ResNet, and VGG (Abdusalomov et al., 2023). As Detectron2 integrates various commonly used deep learning models designed for object detection and instance segmentation, its capabilities suggest the potential for adapting to a wider array of agricultural situations in the future.

The prediction results of the fourth stage were manually confirmed and were more than 99% accurate. Hence, the prediction results of the fourth stage were used as a reference to compare the prediction results of models at different stages using the Jaccard similarity index. The similarity index ranges from 0 to 1, where values closer to 1 indicate the highest similarity. For Detectron2, the similarity index values of the first, second, and third models compared to the fourth model were 0.87, 0.92, and 0.98, respectively. The similarity index of 0.98 between the third and fourth stages indicates very high similarity between the prediction results of these models.

**Figure 8:**
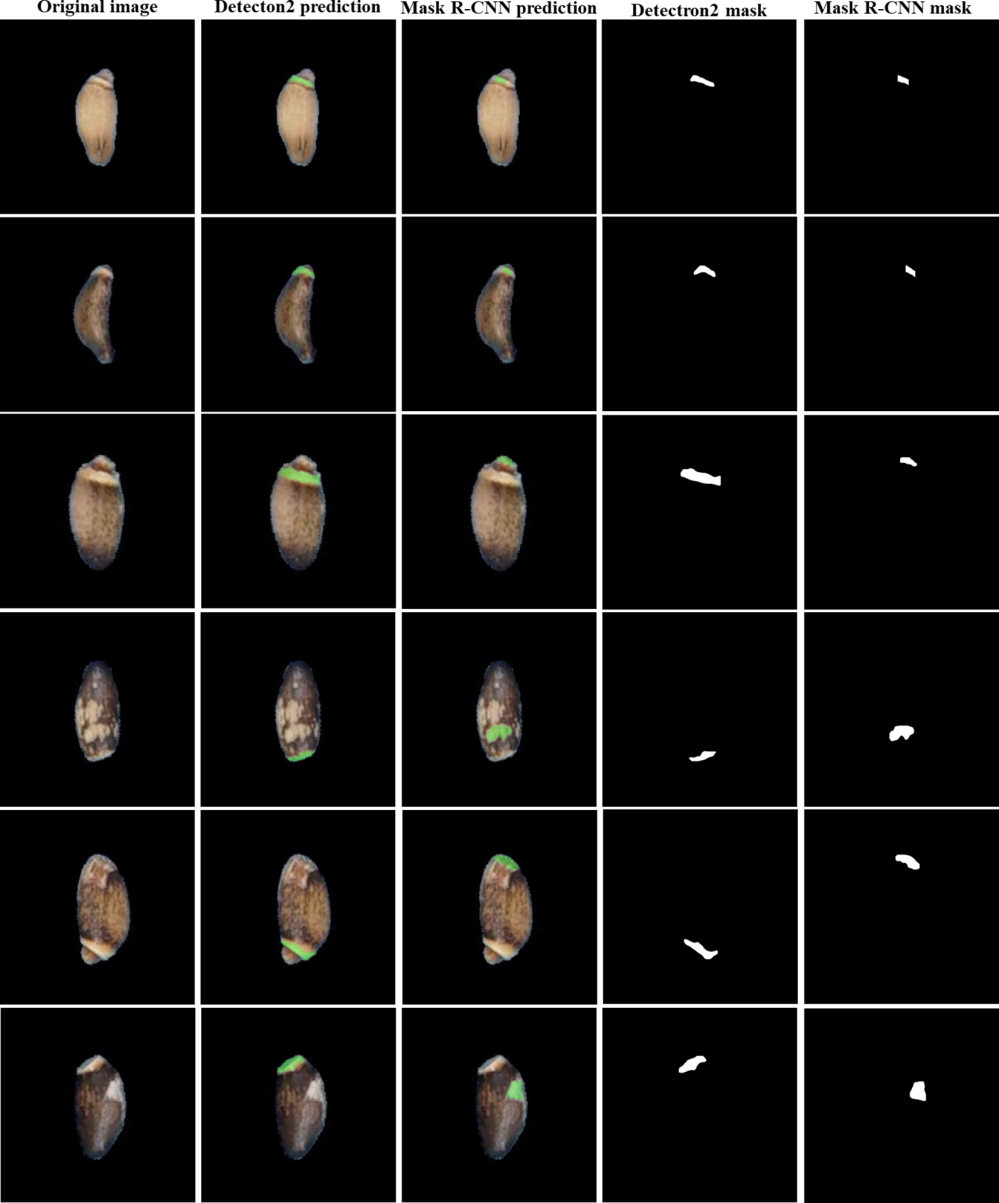
Comparison of elaiosome prediction disagreements between Detectron2 and Mask R-CNN.

The Jaccard similarity index for Mask R-CNN was calculated using the prediction results of Detectron2’s fourth stage as a reference. A Jaccard similarity index of 0.88 between the segmentation results of Detectron2 and Mask R-CNN at the fourth stage indicates a relatively high level of agreement between the segmentation masks produced by the two models. This means that both models are consistent in their segmentation results, with only 12% of the union consisting of images that are not mutually agreed upon (**Table 3**). Although both models showed similar performance in terms of evaluation metrics, manual checking of prediction results confirmed that Detectron2 produces more precise segmentations and is more reliable for elaiosome segmentation than Mask R-CNN (**Figure 8**).

**Table 3:** Jaccard similarity index for different training stages of Detectron2 and Mask R-CNN.

Algorithm	Training stage			
	1st	2nd	3rd	4th
Detectron2	0.87	0.92	0.98	–
Mask R-CNN	0.42	0.57	0.74	0.88

### 3.3 High-throughput phenotyping of various morphological traits in milk thistle seed

Seed morphological traits were analyzed for 397 milk thistle germplasm. We employed our previously developed image-based high-throughput phenotyping method using ImageJ (Baek et al., 2020) to analyze seed morphological traits of milk thistle. Ten seed phenotypes including area, perimeter, major axis, minor axis, solidity, circularity, roundness, solidity, AR, elaiosome area, and the ratio of elaiosome area were measured for a total of ~39,700 seeds using image-based high-throughput phenotyping. In this study, new quantifiable phenotypes of milk thistle seeds, namely, the measurement of elaiosome area and the ratio of elaiosome to the total seed area, were successfully added. Manual measurement and conventional image analysis were not able to extract these phenotypes, indicating the power of deep learning applications in plant phenomics.

**Figure 9:**
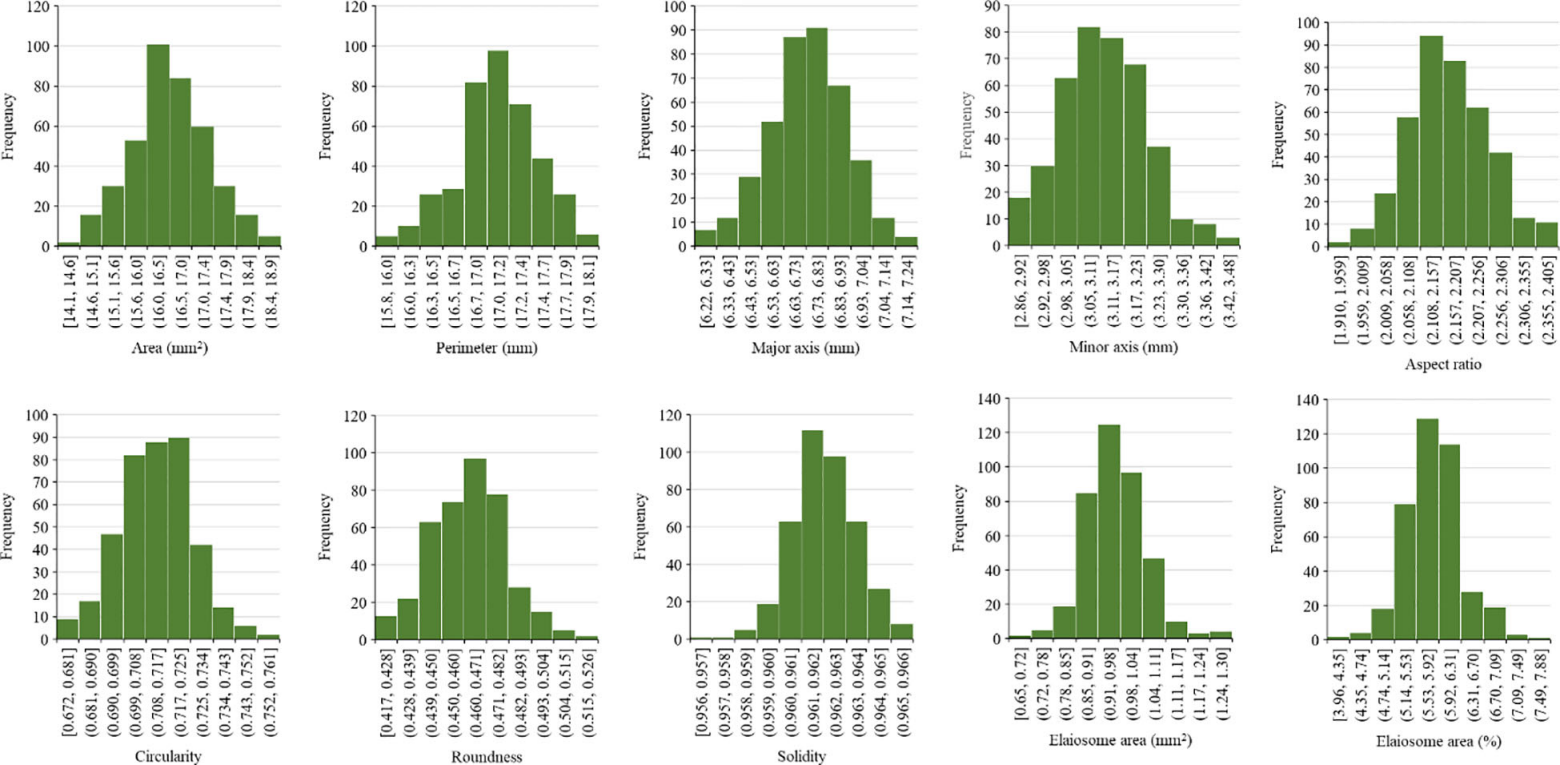
Frequency distribution of 397 milk thistle germplasm based on seed morphological traits (area, perimeter, major axis, minor axis, aspect ratio, circularity, roundness, solidity elaiosome area, and percent elaiosome area). The trait measurements were extracted using ImageJ. The x-axis shows the trait measurement value, and the y-axis shows the number of milk thistle lines.

One hundred seeds were analyzed for each line, and an average was calculated. The frequency distribution of 397 milk thistle germplasms for 10 seed morphological traits of milk thistle is presented in **Figure 9**. The measured traits showed continuous variation and normal distribution. The summary statistics including range, mean, and standard deviation for the 10 morphological traits are presented in **Table 4**. Image-based high-throughput phenotyping is becoming an integral part of plant science studies to measure morphological, physiological, biochemical, and stress response traits in economically important crops (Sun et al., 2022; Abebe et al., 2023). The seed phenotypic data produced in this study can be used for breeding milk thistle cultivars with desirable traits.

**Table 4:** Summary of the descriptive statistics of seed morphological traits for 397 milk thistle germplasm.

	Area	Perimeter	Major axis	Minor axis	Circularity	AR	Roundness	Solidity	Elaiosome area	Elaiosome area %
Average	16.517	17.060	6.740	3.117	0.712	2.170	0.463	0.962	0.962	5.824
Max.	18.859	18.124	7.238	3.482	0.761	2.405	0.526	0.966	1.303	7.878
Min.	14.143	15.786	6.224	2.859	0.672	1.910	0.417	0.956	0.651	3.959
Stdev.	0.815	0.420	0.178	0.114	0.014	0.087	0.019	0.001	0.087	0.497

AR, aspect ratio.

Digital seed phenotyping represents a significant advancement in precision agriculture, offering a more efficient and effective way to enhance crop production and sustainability. The advantages of our approach include reducing labeling time by enabling semi-automatic labeling and reducing the learning time using a small number of training data. Integrating a deep learning-based elaiosome detection method into existing phenotyping workflows has the potential to revolutionize agricultural practices by enhancing efficiency, accuracy, and scalability. This can be achieved by setting up imaging equipment in the laboratory for collecting images of seeds and implementing image preprocessing steps to enhance image quality before feeding them into the deep learning model. Then, the images can be uploaded to the local server, cloud server, or edge device where the elaiosome detection model can run. The detailed seed phenotypic data can be linked with other relevant data such as genomic information and metabolic profile. Investigation of relationships between various seed phenotypic traits with genomic and metabolic information will be the focus of future work, and we are working in collaboration with genomic and metabolic engineering divisions at the National Institute of Agricultural Sciences.

Addressing potential limitations such as the need for manual checking of model predictions and real-time processing limitations is the focus of future research. Furthermore, we aspire to develop a fully automated elaiosome labeling and detection method in the future. Future research should also focus on extending the method to other crops beyond milk thistle, demonstrating its applicability and scalability across different species.

## 4 Conclusions

Our study presents a successful application of the Detectron2 deep learning model for the automated detection and segmentation of elaiosome in milk thistle seed. The developed method uses few manually labeled images for training the first detection model and retrains the model using correctly predicted images, thereby reducing the cost of manually labeling a large number of training data. The developed model exhibits high accuracy (99.9%) of detection and precisely produced segmentation masks of elaiosome. The mask images of elaiosome were used as input for image-based analysis of elaiosome phenotypes in ImageJ. This enabled the successful addition of new quantifiable traits to milk thistle seed phenotype, namely, the elaiosome area and the ratio of elaiosome. Seed morphological traits of 397 milk thistle lines (39,700 seeds) including elaiosome area were accurately analyzed by high-throughput method using image analysis. The findings of this study will open avenues for innovative research in milk thistle seed and offer promising solutions for automating labor-intensive tasks in plant phenotyping studies.

## Data availability statement

The original contributions presented in the study are included in the article. Further inquiries can be directed to the corresponding author.

## Author contributions

YK: Conceptualization, Formal analysis, Investigation, Methodology, Software, Writing – review & editing. AA: Data curation, Writing – original draft, Writing – review & editing, Visualization. JK: Formal analysis, Methodology, Software, Writing – review & editing. SH: Resources, Writing – review & editing. KA: Resources, Writing – review & editing. JS: Resources, Writing – review & editing. JB: Conceptualization, Funding acquisition, Project administration, Writing – review & editing.
